# BEX2 has a functional interplay with c-Jun/JNK and p65/RelA in breast cancer

**DOI:** 10.1186/1476-4598-9-111

**Published:** 2010-05-19

**Authors:** Ali Naderi, Ji Liu, Luke Hughes-Davies

**Affiliations:** 1 The University of Queensland Diamantina Institute, Princess Alexandra Hospital, Brisbane Qld 4102, Australia; 2 Hutchison-MRC Research Centre, Department of Oncology, University of Cambridge, Hills Road, Cambridge CB2 0XZ, UK

## Abstract

**Background:**

We have previously demonstrated that BEX2 is differentially expressed in breast tumors and has a significant role in promoting cell survival and growth in breast cancer cells. BEX2 expression protects breast cancer cells against mitochondrial apoptosis and G1 cell cycle arrest. In this study we investigated the transcriptional regulation of BEX2 and feedback mechanisms mediating the cellular function of this gene in breast cancer.

**Results:**

We found a marked induction of BEX2 promoter by c-Jun and p65/RelA using luciferase reporter assays in MCF-7 cells. Furthermore, we confirmed the binding of c-Jun and p65/RelA to the BEX2 promoter using a chromatin immunoprecipitation assay. Importantly, transfections of c-Jun or p65/RelA in MCF-7 cells markedly increased the expression of BEX2 protein. Overall, these results demonstrate that BEX2 is a target gene for c-Jun and p65/RelA in breast cancer. These findings were further supported by the presence of a strong correlation between BEX2 and c-Jun expression levels in primary breast tumors. Next we demonstrated that BEX2 has a feedback mechanism with c-Jun and p65/RelA in breast cancer. In this process BEX2 expression is required for the normal phosphorylation of p65 and IκBα, and the activation of p65. Moreover, it is necessary for the phosphorylation of c-Jun and JNK kinase activity in breast cancer cells. Furthermore, using c-Jun stable lines we showed that BEX2 expression is required for c-Jun mediated induction of cyclin D1 and cell proliferation. Importantly, BEX2 down-regulation resulted in a significant increase in PP2A activity in c-Jun stable lines providing a possible underlying mechanism for the regulatory effects of BEX2 on c-Jun and JNK.

**Conclusions:**

This study shows that BEX2 has a functional interplay with c-Jun and p65/RelA in breast cancer. In this process BEX2 is a target gene for c-Jun and p65/RelA and in turn regulates the phosphorylation/activity of these proteins. These suggest that BEX2 is involved in a novel feedback mechanism with significant implications for the biology of breast cancer.

## Introduction

We have previously demonstrated that BEX2, a member of Brain Expressed X-linked gene family, is differentially expressed in breast tumors and BEX2 expression predicts the response to tamoxifen therapy [1]. Although BEX2 shows a relatively higher expression in 15% of breast cancers, this gene is expressed in the majority of breast tumors and breast cancer cell lines [1,2]. The BEX genes were originally found to have a developmental function and a role in the neurological diseases such as accumulation in retinal ganglion cells after optic nerve stroke [3,4]. However, recent studies strongly suggest their involvement in cancer biology. For example BEX1 is overexpressed in neuroendrocrine tumors and is down-regulated in glioblastoma cells compared to normal tissue [5,6]. BEX3 is shown to be expressed in teratocarcinoma cells, is associated with the mitochondria, and is required for cell cycle entry in these cancer cells [7]. In addition to our data in breast cancer, BEX2 is found to be differentially expressed in acute myeloid leukemia with a higher expression observed in MLL subtype [8]. It has been reported that BEX2 is a binding partner of LMO2, a T-cell oncogene with recurrent chromosomal translocations in T-cell acute leukemias [9], and enhances the transcriptional activity of LMO2-NSCL2 complex [10]. Furthermore, in AML and glioblastomas BEX2 expression is regulated by epigenetic mechanisms such as promoter methylation [6,8]. However, we have not found any correlation between BEX2 expression and promoter methylation in breast tumors or any evidence for gene amplification to explain the differential expression of BEX2 in breast cancer [1]. These suggest that disturbances in transcriptional regulation may be a mechanism for the observed pattern of BEX2 expression in breast cancer.

Moreover, we have demonstrated that BEX2 has a significant role in promoting cell survival and growth in breast cancer cells [1,2]. BEX2 down-regulation induces mitochondrial apoptosis and sensitizes breast cancer cells to pro-apoptotic agents and conversely, BEX2 overexpression protects these cells against mitochondrial apoptosis [1,2]. In addition, we have shown that this effect of BEX2 is mediated through the modulation of Bcl-2 protein family, including the regulation of Bcl-2 and BAD phosphorylation [2]. Furthermore, our data suggest that BEX2 expression is required for the normal cell cycle progression during G1 in breast cancer cells through the regulation of cyclin D1 [2]. Importantly, we have shown that BEX2 down-regulation results in a higher activity of Protein Phosphatase 2A (PP2A), [2]. The modulation of PP2A, which is known to regulate several key proteins involved in mitochondrial apoptosis and G1 cell cycle [11,12], provides a possible mechanism to explain the BEX2-mediated cellular effects.

In this study we investigate the mechanism of transcriptional regulation of BEX2 and demonstrate that the BEX2 gene is a target of c-Jun and p65/RelA transcription factors. Furthermore, we show that BEX2 is necessary for the phosphorylation of c-Jun/JNK and p65 in breast cancer cells. This study suggests that BEX2 has a functional interplay with c-Jun/JNK and p65, which has significant implications for the biology of breast cancer.

## Results

### BEX2 expression is regulated by ceramide and IкBα phosphorylation

In order to investigate the transcriptional regulation of BEX2 we first investigated the factors involved in the regulation of BEX2 expression. We have previously observed that ceramide and Nerve Growth Factor (NGF) treatments induce BEX2 expression in MCF-7 cells [1]. To further investigate these findings we studied the effects of NGF, the IкBα phosphorylation inhibitor BAY11-7085 (BAY11), overexpression of IκBα Dominant-Negative (DN), and ceramide on BEX2 expression using MCF-7 and MDA-MB-231 cell lines. We confirmed the activity of BAY11 inhibitor by demonstrating inhibition of IкBα phosphorylation with an ELISA assay (data not shown). BEX2 expression was measured using Real-Time PCR (RT-PCR).

**Figure 1:**
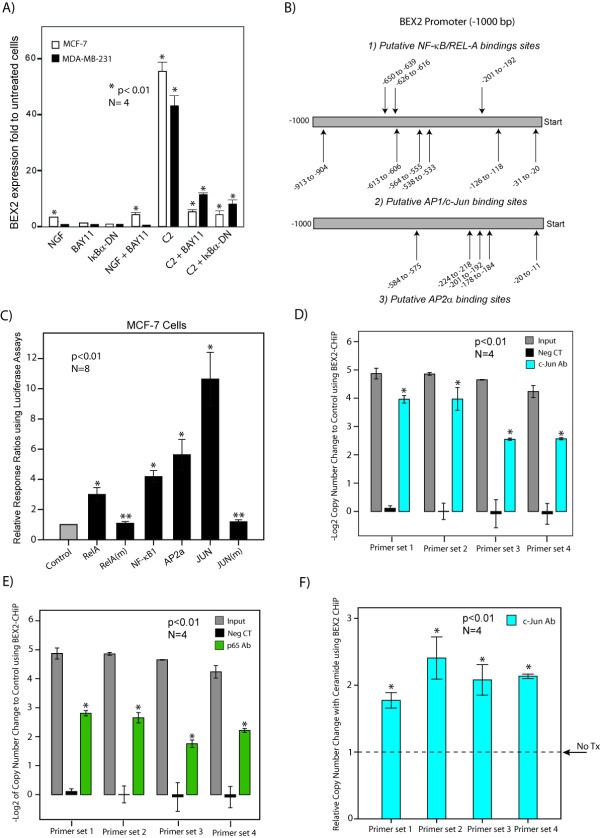
**Real-Time PCR, Reporter Assay and ChIP to identify BEX2 transcription factors. **(A) BEX2 expression folds using RT-PCR in MCF-7 and MDA-MB-231 cell lines after treatments with NGF at 200 ng/ml, BAY11-7085 (BAY11) at 5 μM, and C2 at 10 μM. Expression was measured relative to the untreated cells. The effect of IκBα dominant-negative (DN) on BEX2 expression was assessed using the transfection of an IκBα-DN construct. Forty-eight hours after the transfection, C2 treatment was carried out at 10 μM overnight. BEX2 expression fold was measured relative to the untreated cells transfected with an empty vector. *, is compared to untreated cells. Error Bars: ± 2SEM. (B) Putative transcription factor binding sites for NF-κB/RelA, AP1/c-Jun and AP2α in 1 kb promoter region of BEX2. The locations of putative binding site are demonstrated by arrows. (C) Luciferase reporter assays. The transcriptional activities of p65/RelA, p50/NF-κB1, AP2α, c-Jun, c-Jun mutant (Ser63→Ala), and p65/RelA mutant (Ser468→Ala) expression constructs were measured using Dual-Luciferase Assays in MCF-7 cells (m: mutant construct). The *Renilla *pRL-TK vector was used as an internal control reporter. Transfections with BEX2 reporter vector only and empty pcDNA vector were used as a control. The response ratios are demonstrated relative to the internal control reporter (relative response ratio). *, is compared to the control group. **, is mutant c-Jun or p65 compared to the wild type protein. Error Bars: ± 2SEM. (D) ChIP assay with c-Jun antibody. The results of end point RT-PCR amplification using SYBR green method are demonstrated for chromatin immunoprecipitation (ChIP) assays with four sets of primers for BEX2 promoter. Rabbit polyclonal c-Jun antibody was applied at 1:50 dilution. Amplification of input chromatin at a dilution of 1:100 prior to immunoprecipitation was used as a positive control and ChIP using non-specific antibody (rabbit IgG) and distant primer sets (5 kb) served as negative controls. Copy number changes of end point RT-PCR amplification are shown as -Log2 value for each experimental set. *, is compared to the negative control. Error Bars: ± 2SEM. (E) ChIP assay with p65 antibody. The results of end point RT-PCR amplification for ChIP assay using a ChIP-grade rabbit polyclonal p65 antibody at 1:100 dilution as explained in (D). (F) ChIP assay with c-Jun antibody following ceramide induction. The copy number changes for ChIP assays were measured following the induction with ceramide at 10 μM overnight. Rabbit polyclonal c-Jun antibody was applied at 1:50 dilution as explained in (D). The relative copy number changes using end point RT-PCR amplification are demonstrated for each BEX2-promoter primer set. *, is compared to no ceramide group. Error Bars: ± 2SEM.

We observed that ceramide markedly increased BEX2 expression by 40 to 60-fold in MCF-7 and MDA-MB-231 cell lines (p < 0.01, Figure 1A). Furthermore, both BAY11 treatment and overexpression of IκBα-DN almost completely reversed this effect of ceramide on BEX2 expression (p < 0.01, Figure 1A). It is notable that NGF only slightly induced BEX2 expression in MCF-7, while BAY11 treatment or IκBα-DN alone did not have any significant effect (Figure 1A). Furthermore, other pro-apoptotic models such as BAY11 at 7 μM, serum starvation, and tamoxifen treatment at 10 μM did not change the expression of BEX2 (data not shown), indicating that the observed effect with ceramide is not a non-specific transcriptional effect of apoptosis. These findings demonstrate that ceramide has a striking regulatory effect on BEX2 expression in breast cancer cells and IкBα phosphorylation is necessary for a full response.

### BEX2 is a c-Jun and p65 target gene

To identify the transcription factors that regulate BEX2 expression and involved in the biological functions of this gene, we first assessed BEX2 promoter for candidate transcription factor binding sites using bioinformatics programs (see methods). Analysis of binding sites in the 1 kb promoter region of BEX2 was carried out using PATCH™ public 1.0 software and the TRANSFAC^® ^6.0 data base. We identified six AP-1/c-Jun candidate binding sites, three NF-κB/RelA sites, and five AP2α sites (Figure 1B). These observations are important since both c-Jun and AP2 are known to mediate the transcriptional activation of ceramide signaling [13,14].

We next used a dual-luciferase reporter assay to examine the effects of the predicated transcription factors on the regulation of BEX2 promoter. For this purpose we cloned and sequenced the 1.2 kb promoter region of BEX2 in a pGL3 luciferase reporter vector (Promega). Expression constructs for c-Jun, p65/RelA, p50/NF-κB1, and AP2α were cloned and sequenced in pcDNA™3.1 vector (Invitrogen). Mutant constructs of c-Jun (Ser63→Ala) and p65 (Ser468→Ala) were generated as described in methods. MCF-7 cells were co-transfected with the BEX2 reporter vector and each of the transcription factors or mutant constructs. The *Renilla *pRL-TK vector was used as an internal control reporter. Co-transfection with the BEX2 reporter vector and the empty pcDNA vector were used as the control. Forty-eight hours after the transfections reporter activity was measured with the Dual-Glo™ Luciferase Assay System (Promega). Next, the response ratios for transcription factors and control were measured relative to the internal control reporter (relative response ratio). We observed a marked increase in BEX2 reporter activity with c-Jun by approximately 11-fold (p < 0.01, Figure 1C). Furthermore, RELA, NF-κB1, and AP2α significantly increased BEX2 reporter activity by approximately 2.7 to 5-fold (p < 0.01, Figure 1C). The control transfection resulted in a relative ratio close to 1 (Figure 1C). In addition, mutant constructs of c-Jun (Ser63→Ala) and p65 (Ser468→Ala) lacked the ability to induce the BEX2 promoter (Figure 1C). These findings suggest that c-Jun, NF-κB genes, and AP2α significantly activate BEX2 promoter in breast cancer cells.

To further validate the reporter assay findings we tested c-Jun and p65/RelA binding to BEX2 promoter in MCF-7 cells using chromatin immunoprecipitation (ChIP) assays with ChIP-validated c-Jun and p65 antibodies (see methods). Four sets of primers for BEX2 promoter were used for the end point RT-PCR amplification using SYBR green method (Applied Biosystems). These primers were quality controlled using PCR amplification of MCF-7 genomic DNA followed by Agarose gel electrophoresis and sequencing (Additional file 1, Figure S1). Amplification of input chromatin at a dilution of 1:100 prior to immunoprecipitation served as a positive control for ChIP assays and ChIP using non-specific antibody (rabbit IgG) and distant primer sets (5 kb) served as negative controls. ChIP experiments were carried out with and without ceramide induction at 10 μM concentration overnight. Copy number changes were calculated as -Log2 value for each experimental set (Figure 1D-F). We observed significant enrichments for the BEX2 promoter region with c-Jun and p65 antibodies, a result was seen with each of the four primer sets (Figure 1D and 1E). These enrichments were approximately 6 to 16-fold and 4 to 8-fold for c-Jun and p65, respectively (p < 0.01, Figure 1D and 1E). It is notable that we observed a further 2-fold increase in this enrichment following ceramide induction using c-Jun antibody, which was also reproducible with all primer sets (p < 0.01, Figure 1F). This increase, which was not observed with p65 antibody, suggests that c-Jun activation is involved in the induction of BEX2 with ceramide treatment. Overall these data demonstrate that BEX2 is a target gene for c-Jun and p65/RelA in breast cancer cells.

Moreover, we carried out ChIP assays with c-Jun and p65 antibodies following the transient transfections of MCF-7 cells with either wild type c-Jun and p65/RelA or the mutant constructs of c-Jun (Ser63→Ala) and p65 (Ser468→Ala). Transfection with an empty vector was used as a control. ChIP assays were carried out forty-eight hours after the transfections and the enrichment of BEX2 promoter region was assessed using the end point RT-PCR amplification. We observed 8 to 16-fold enrichments with p65 and c-Jun antibodies, respectively following transfections with the wild type constructs (Additional file 2, Figure S2). However, we did not observe any significant enrichment for BEX2 promoter following transfections with the mutant constructs of c-Jun (Ser63→Ala) and p65 (Ser468→Ala), suggesting that these mutants act as a dominant negative and are not capable of binding to the BEX2 promoter region (Additional file 2, Figure S2). Therefore, in order to bind and activate BEX2 promoter, c-Jun and p65 require phosphorylation at Ser63 and Ser468 sites, respectively.

### c-Jun and p65 induce BEX2 protein expression

**Figure 2:**
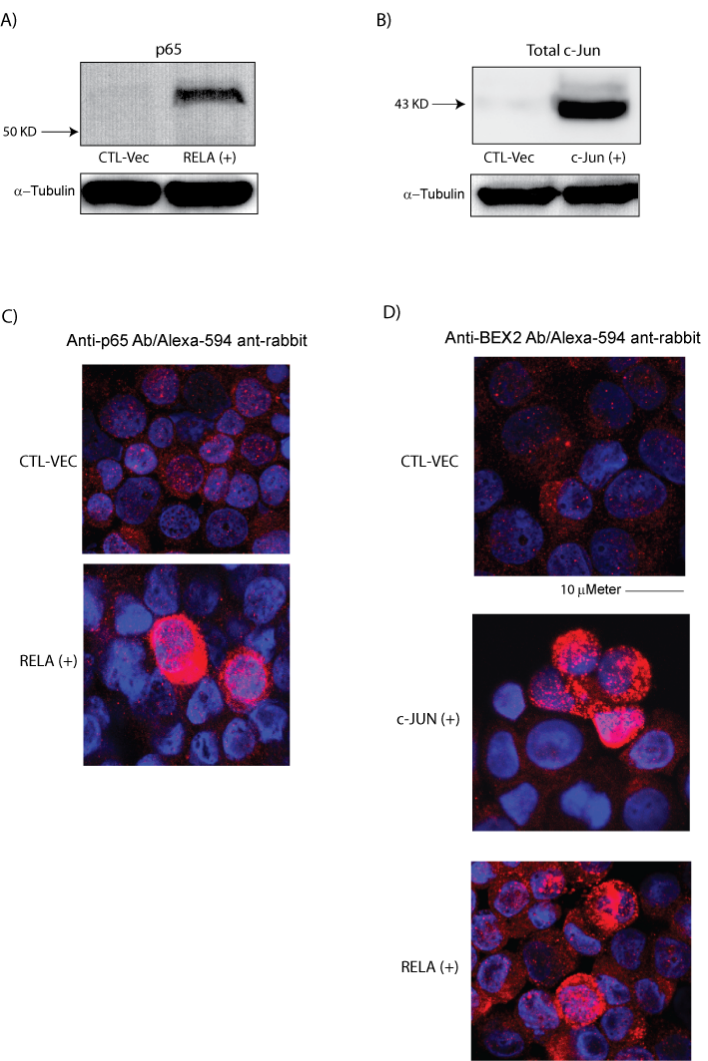
**The induction of BEX2 protein expression following p65 and c-Jun transfections. **(A) Western blot analysis of p65. MCF-7 cell line was transfected with p65/pcDNA3.1 (RELA(+)) or an empty vector (CTL-VEC). The overexpression of p65/RelA was confirmed 48 h after the transfection by western blot analysis using p65 rabbit monoclonal antibody at 1:500 dilution. (B) Western blot analysis of c-Jun. MCF-7 cell line was transfected with c-Jun/pcDNA3.1 (c-Jun(+)) or an empty vector (CTL-VEC). The overexpression of c-Jun was confirmed 48 h after the transfection by western blot analysis using c-Jun rabbit monoclonal antibody at 1:1000 dilution. (C) Immunofluorescence staining of p65. MCF-7 cell line was transfected with p65/pcDNA3.1 (RELA(+)) or an empty vector (CTL-VEC: top panel). Immunofluorescence staining demonstrates p65/RelA overexpression (bottom panel) using anti-p65 primary and Alexa-594 anti-rabbit secondary antibodies at 1:200 and 1:500 dilutions, respectively. (D) Immunofluorescence staining of BEX2 following p65 and c-Jun overexpression. IF staining was carried out 48 h after transfections with c-Jun (c-Jun(+): middle panel) or p65/RelA (RELA(+): bottom panel) using a rabbit polyclonal BEX2 antibody and Alexa-594 secondary antibody at 1:100 and 1:500 dilutions, respectively. An empty vector was used as the control (CTL-VEC: top panel).

To further investigate the effects of c-Jun and p65/RelA on the regulation of BEX2 expression, we assessed changes in the BEX2 protein level following the overexpression of c-Jun and p65/RelA. Transient transfections of c-Jun and p65/RelA constructs were separately performed in MCF-7 cells and transfection with an empty vector was used as a control. The overexpression of c-Jun and p65 were confirmed 48 h after the transfections by western blot analysis using p65 rabbit polyclonal (Abcam) and rabbit c-Jun monoclonal (Cell Signaling) antibodies (Figure 2A and 2B). We also confirmed the overexpression of p65 by immunofluorescence (IF) using anti-p65 primary and Alexa-594 anti-rabbit secondary (Invitrogen) antibodies (Figure 2C). To assess the effects of c-Jun and p65/RelA overexpression on BEX2 protein level, IF staining was carried out 48h after transfections using a rabbit polyclonal BEX2 antibody, that we have previously described [2], and Alexa-594 secondary antibody. Notably, we observed a significant increase in BEX2 protein expression in the transfected cells compared to the control and untransfected neighboring cells following both c-Jun and p65 overexpression experiments (Figure 2D). These findings demonstrate that c-Jun and p65 induce BEX2 protein expression and further support that the BEX2 promoter is targeted by c-Jun and p65.

### BEX2 expression enhances p65 nuclear transport

The fact that BEX2 transcription is strongly regulated by c-Jun and p65 suggests that BEX2 may have a role in the cellular activities mediated by these proteins. Furthermore, we have previously demonstrated that BEX2 expression is necessary for the NGF-mediated activation of NF-κB in breast cancer cells and found that p65-nuclear staining, as a measure of NF-κB activation, is approximately 2-fold higher in breast tumor samples with a relative overexpression of BEX2 [1,2].

**Figure 3:**
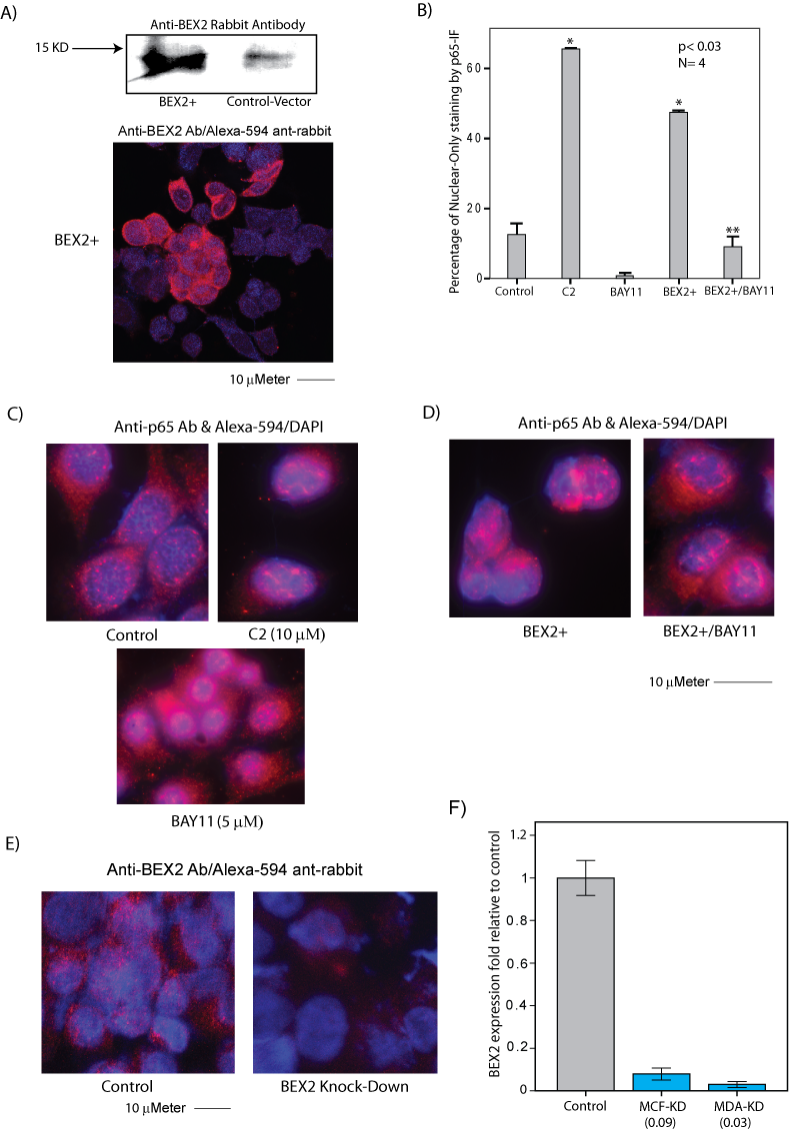
**Immunofluorescence to assess p65 nuclear localization, and validation of BEX2 overexpression and knock-down. **(A) Western blot analysis and immunofluorescence (IF) to confirm BEX2 overexpression. MCF-7 cells were transfected with either a BEX2 expression construct or control vector. Forty-eight hours after transfections, BEX2 overexpression was assessed by western blot analysis using BEX2 rabbit polyclonal antibody at 1:200 dilution (top panel) and by IF using BEX2 antibody at 1:100 dilution (bottom panel). For IF staining Alexa-594 anti-rabbit secondary antibody was applied at 1:500 dilution. (B) The percentage of Nuclear-Only staining for p65 by IF in MCF-7 cells. Forty-eight hours after transfections cell treatments were carried out in the following groups: 1) control-vector, 2) control-vector + ceramide (C2) treatment at 10 μM overnight, 3) control-vector + BAY11-7082 (BAY11) at 5 μM overnight, 4) BEX2 overexpression (BEX2+), and 5) BEX2 overexpression + BAY11 treatment overnight. The following day, IF staining was carried out using anti-p65 primary and Alexa-594 anti-rabbit secondary antibodies at 1:200 and 1:500 dilutions, respectively. *, is for C2 or BEX2+ group vs control and **, is for BEX2+/BAY11 vs BEX2+. Error Bars: ± 2SEM. (C) Cellular localization of p65 by IF in the control, ceramide-treated, and BAY11-treated MCF-7 cells as explained in (B). (D) Cellular localization of p65 by IF following BEX2 overexpression (BEX2+) with and without BAY11 treatment as explained in (B). (E) BEX2 protein level by IF after BEX2 Knock-Down in MCF-7 cells. Anti-BEX2 rabbit primary and anti-rabbit Alexa-594 secondary antibodies were used at 1:100 and 1:500 dilutions, respectively. Left panel: control, right panel: BEX2 knock-down. (F) BEX2 knock-down (KD) efficiencies by RT-PCR for BEX2-siRNA duplexes in breast cancer cell lines MCF-7 (MCF-KD) and MDA-MB-231 (MDA-KD). BEX2 transcript level following knock-down was measured relative to the non-targeting siRNA control. The average fold changes for the two sets of siRNA duplexes are shown in each cell line. Error Bars: ± 2SEM.

To further investigate the role of BEX2 in p65 activation we assessed the nuclear localization of p65 following BEX2 overexpression. The activation of p65 following phosphorylation results in nuclear translocation and DNA binding of this protein [15]. Furthermore, an inhibition of IκBα phosphorylation inactivates p65 and other NF-κB proteins [16]. BEX2 overexpression was carried out in MCF-7 cells using a BEX2-expression vector as described before [2]. Overexpression of BEX2 was confirmed 48 h after the transfection by western blot analysis and IF using rabbit polyclonal BEX2 antibody (Figure 3A). An empty vector was used as a control for these experiments. Forty-eight hours after transfections cells were treated in the following groups overnight: 1) control vector (no treatment), 2) control vector + ceramide at 10 μM (positive control), 3) control vector + BAY11 at 5 μM (negative control), 4) BEX2-vector, and 5) BEX2-vector + BAY11 at 5 μM. IF experiments were carried out the following day using primary anti-p65 and secondary Alexa-594 antibodies. The percentage of cells with only nuclear staining of p65 (activated p65) were measured and compared between different treatment groups. As expected the percentage of nuclear-only p65 staining was significantly increased with ceramide treatment and decreased with BAY11 (Figure 3B and 3C). Importantly, BEX2 overexpression resulted in a 3-fold increase in the percentage of nuclear-only p65 staining (p < 0.01, Figure 3B and 3D left panel) and this effect was completely reversed with the addition of BAY11 (Figure 3B and 3D right panel). These data suggest that BEX2 overexpression increases the nuclear localization of p65 and IκBα phosphorylation is necessary for this effect.

### BEX2 regulates p65 phosphorylation and activation

**Figure 4:**
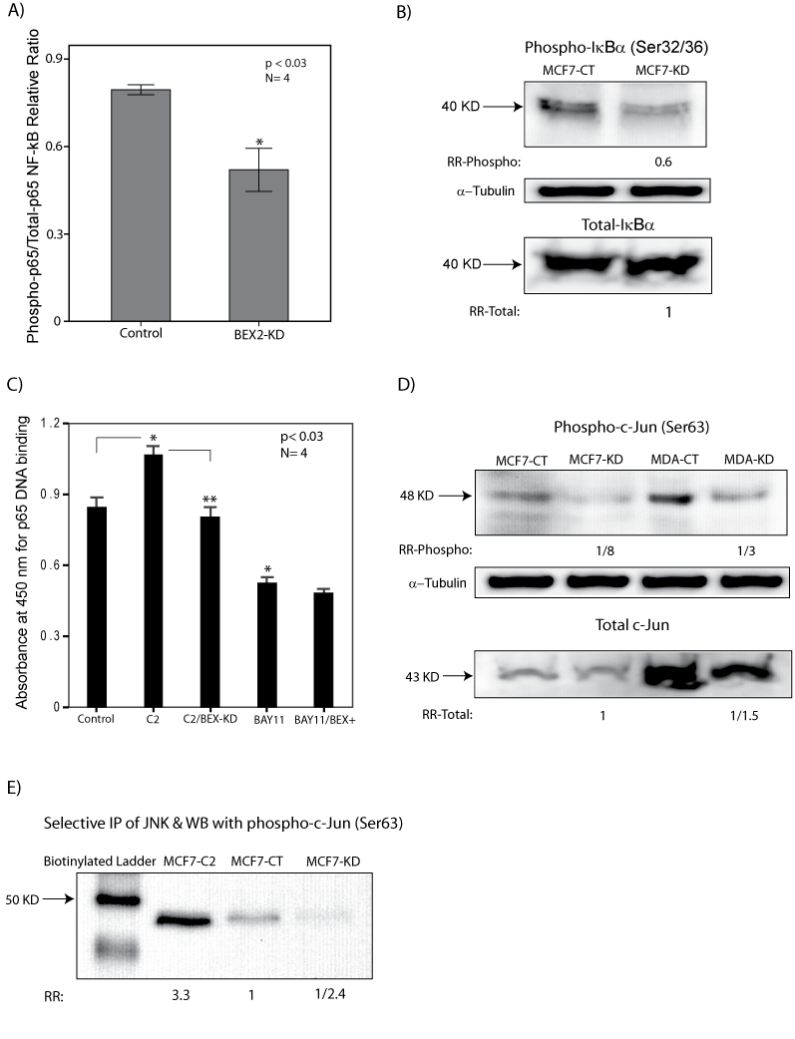
**The effect of BEX2 expression on the phosphorylation of p65, IκBα, c-Jun, and JNK Kinase activity. **(A) Phospho-p65 level using ELISA. The levels of phospho-p65 (Ser468) and total-p65 were measured using ELISA after transfections with either control-siRNA or BEX2-siRNA in MCF-7 cells. Relative ratio for phospho-p65/total-p65 is shown for each group. *, is for BEX2-KD vs control experiments. Error Bars: ± 2SEM. (B) Phospho-IкBα level by western blot analysis. The levels of phospho-IкBα and total-IкBα were measured by western blot analysis after transfections with either control-siRNA (CT) or BEX2-siRNA (KD) in MCF-7 cells. IκB-α rabbit polyclonal and phospho-IκB-α (Ser32/36) mouse monoclonal antibodies were used at 1:1000 dilutions. Rabbit polyclonal α-tubulin was used as the loading control. Fold changes (RR) in band densities following BEX2-KD were measured relative to the control group (CT-siRNA). (C) Measurement of p65 DNA binding in MCF-7 cells using ELISA. The measurements were carried out in the following groups; 1) control: control-siRNA, 2) C2: control-siRNA + ceramide treatment at 10 μM ON, 3) C2/BEX-KD: BEX2-siRNA + ceramide, 4) BAY11: control-vector + BAY11 at 5 μM ON, and 5) BAY11/BEX2+: BEX2-vector + BAY11. *, is for ceramide or BAY11 group vs control; **, is for ceramide group vs ceramide + BEX2-KD. Error Bars: ± 2SEM. (D) Phospho-c-Jun level by western blot analysis. The levels of phospho-c-Jun (Ser63) and total-c-Jun were measured by western blot analysis after transfections with either control-siRNA (CT) or BEX2-siRNA (KD) in MCF-7 and MDA-MB-231 cell lines. Total-c-Jun rabbit monoclonal and phospho-c-Jun (Ser63) rabbit monoclonal antibodies were used at 1:1000 dilutions. Fold changes (RR) in band densities following BEX2-KD were measured relative to the control group (CT-siRNA). (E) JNK Kinase activity. JNK kinase assay was carried out using a selective immunoprecipitation of JNK followed by JNK kinase assay and western blot for phospho-c-Jun (Ser63). JNK kinase activities were measured after transfections with either control-siRNA (CT) or BEX2-siRNA (KD) in MCF-7 cells. Ceramide (C2) treatment at 10 μM overnight was used as a positive control for JNK induction. Fold changes (RR) in band densities following ceramide treatment and BEX2-KD were measured relative to the control group (CT-siRNA).

To explain the observed effect of BEX2 on p65 nuclear transport, we next investigated whether BEX2 expression regulates the phosphorylation of p65 or IκBα. To examine these we assessed the effect of BEX2 knock-down (KD) on the phosphorylation of p65 and IκBα in MCF-7 cells. BEX2-KD was carried out using siRNA oligos (duplex) as we previously published [2]. Two sets of BEX2-siRNA duplexes were used for BEX2-KD and non-targeting siRNA was used as a control. All the knock-down experiments were carried out using each BEX2-siRNA duplex and the quantitative data presented for each experiment is the average result obtained from the two BEX2 siRNA-duplexes. The down regulation of BEX2 protein after BEX2-KD was confirmed using IF with anti-BEX2 antibody (Figure 3E). In addition, using RT-PCR we observed more than 90% reduction in BEX2 transcript following BEX2-KD (Figure 3F). We subsequently examined the effect of BEX2 down-regulation on the baseline phosphorylation level of p65 (Ser468) in MCF-7 cells using ELISA. There was a modest but significant reduction in phospho-p65/total-p65 ratio by 0.65-fold following BEX2-KD (p < 0.03, Figure 4A). Furthermore, we observed a similar level of reduction in phospho-IκBα/total-IκBα by 0.6-fold following BEX2-KD using western blot analysis (Figure 4B). To investigate whether BEX2 expression is necessary for the down-stream p65 activation we assessed the p65 DNA binding using ELISA. Ceramide treatment, which is known to activate p65/NF-κB [17], was carried out at 10 μM concentration overnight to induce p65. Notably, we observed that ceramide significantly increased the p65 DNA binding (p < 0.03) and this effect was inhibited by BEX2-KD (Figure 4C). Furthermore, BAY11 at 5 μM significantly reduced the p65 DNA binding and this reduction was not overcome by the overexpression of BEX2 (Figure 4C). Taken together, these findings suggest that BEX2 expression is required for both normal phosphorylation of p65 and IκBα, and the ceramide induced DNA binding of p65 in breast cancer cells.

### BEX2 is necessary for c-Jun phosphorylation and JNK activity

To further investigate a cross-regulation between BEX2 and the transcription factors mediating its expression, we next assessed the effect of BEX2 expression on the phosphorylation of c-Jun (Ser63). BEX2-KD was carried out using siRNA duplexes in MCF-7 and MDA-MB-231 cell lines and non-targeting siRNA was used as a control. The levels of total and phospho-c-Jun were measured and compared between the knock-down and control experiments using western blot analysis. Importantly, we observed a reduction in c-Jun phosphorylation following BEX2-KD by 8-fold in MCF-7 and by 3-fold in MDA-MB-231 cell lines (Figure 4D, fold changes are the average of three replicates).

Since the phosphorylation of c-Jun is regulated by c-Jun-N-terminal Kinase (JNK), [18], we next investigated the effect of BEX2 down-regulation on JNK kinase activity. JNK kinase assay was carried out using a selective immunoprecipitation of JNK with the application of c-Jun-Agarose beads followed by JNK kinase assay and western blot for phospho-c-Jun (Ser63). Experiments were carried out in MCF-7 cell line and ceramide treatment at 10 μM overnight was used as a positive control for JNK induction [19]. BEX2-KD was carried out as described before and non-targeting siRNA was used as a control. We observed a 3.3-fold increase in JNK activity following ceramide treatment (Figure 4E). Moreover, there was a 2.4-fold reduction in JNK kinase activity following BEX2-KD compared to the control (Figure 4E). This finding suggests that BEX2 expression is necessary for c-Jun phosphorylation and JNK kinase activity in breast cancer cells.

### BEX2 expression is required for c-Jun-mediated induction of cyclin D1 and cell proliferation

**Figure 5:**
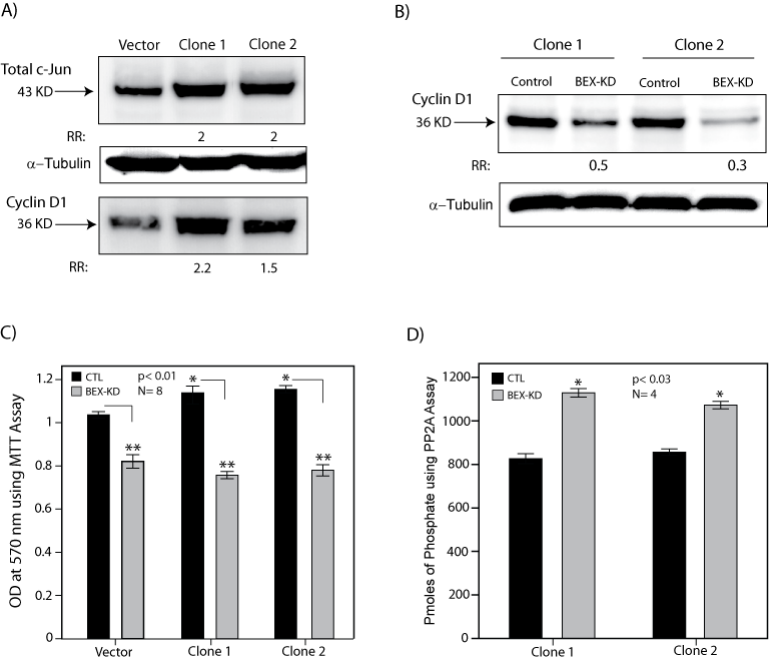
**The effect of BEX2 expression in c-Jun stable lines. **(A) The levels of c-Jun and cyclin D1 in c-Jun stable clones. Stable c-Jun(+) clones (clones 1 and 2) were generated following the transfection of MCF-7 with c-Jun/pcDNA3.1 vector followed by selection in the presence of Geneticin. Transfection with an empty pcDNA vector was used as a control (vector). Western blot analysis for c-Jun and cyclin D1 were carried out using c-Jun and cyclin D1 rabbit monoclonal antibodies at 1:1000 dilutions. Rabbit polyclonal α-tubulin was used as the loading control. Fold changes (RR) in band densities for c-Jun(+) clones 1 and 2 were measured relative to the vector control. (B) The levels of cyclin D1 following BEX2 knock-down. The levels of cyclin D1 was measured by western blot analysis after transfections with either control-siRNA (CT) or BEX2-siRNA (KD) in c-Jun(+) stable clones. Fold changes (RR) in band densities following BEX2-KD were measured relative to the control group. (C) The effect of BEX2 knock-down (KD) on cell proliferation using MTT assay. BEX2-KD was carried out in stable c-Jun(+) clones and vector control (CTL). Absorbance measurements at 570 nM are demonstrated for BEX2-KD and control-siRNA experiments in the stable lines. *, is for clone 1 or 2 vs control vector and **, is for BEX2-KD vs control-siRNA. Error Bars: ± 2SEM. (D) BEX2 regulation of PP2A activity. PP2A immunoprecipitation phosphatase assay in c-Jun(+) clones 1 and 2 after transfections with either control-siRNA (CT) or BEX2-siRNA (KD). Pmoles of phosphate are demonstrated for each group. *, is for BEX2-KD vs control-siRNA. Error Bars: ± 2SEM.

To study the role of BEX2 in c-Jun-mediated cellular functions we first generated stable MCF-7 lines with c-Jun overexpression (c-Jun(+)). A c-Jun/pcDNA3.1 vector was transfected in MCF-7 cells and stable lines were generated using Geneticin (Invitrogen) selection as described in methods. Individual neomycin-resistant colonies were isolated, expanded and analyzed for c-Jun expression using western blot analysis. Transfection with an empty pcDNA vector and following the same process was used as a control. We identified two stable c-Jun(+) clones, which showed a 2-fold overexpression of c-Jun protein (clones 1 and 2, Figure 5A). These clones demonstrated the morphological characteristics of c-Jun overexpression, including growth in a less compact fashion compared to the control cells [20], and irregular shapes with a variable size (Additional file 3, Figure S3A-D). It has been demonstrated that cyclin D1 is a direct c-Jun target gene and is involved in c-Jun-mediated G1 progression [21]. To assess the molecular effects of c-Jun overexpression, we examined the level of cyclin D1 in stable cell lines using western blot analysis. We observed a 1.5 to 2.2-fold increase in the level of cyclin D1 in c-Jun(+) stable lines compared to the vector control (Figure 5A).

To investigate the functional role of BEX2 expression in c-Jun(+) lines, we carried out BEX2-KD using siRNA duplexes as explained before. A non-targeting siRNA was used as a control. Next, the level of cyclin D1 was compared between c-Jun(+)/BEX2-KD and c-Jun(+)/control-siRNA cells. Notably, we observed a marked reduction in cyclin D1 level following BEX2-KD to 0.5 and 0.3-fold of the baseline in clones 1 and 2, respectively (Figure 5B). We next assessed the effect of BEX2 expression on c-Jun-mediated proliferation in c-Jun(+) lines. Cell proliferation was compared between c-Jun(+)/BEX2-KD and c-Jun(+)/control-siRNA lines using MTT assay. A stable vector line was used as the control. We observed a significant increase in cell proliferation in c-Jun(+) clones 1 and 2 compared to the control (p < 0.01, Figure 5C). Importantly, there was a significant reduction in cell proliferation in c-Jun(+) and control lines following BEX2-KD (p < 0.01, Figure 5C). All together, these findings suggest that BEX2 expression is required for c-Jun-mediated induction of cyclin D1 and cell proliferation in breast cancer cells. Furthermore, c-Jun overexpression cannot overcome the effect of BEX2-KD in reduction of cell proliferation.

We have previously shown that BEX2 down-regulation results in a higher PP2A activity in breast cancer cells [2]. Furthermore, it has been demonstrated that the induction of PP2A activity reduces c-Jun phosophorylation and inactivates the transcription of c-Jun-responsive gene cyclin D1 [12]. Therefore, to identify a possible underlying cause for the functional changes observed following BEX2 down-regulation in c-Jun(+) lines we measured the PP2A phosphatase activity using the immunoprecipitation assay. PP2A activity was compared between c-Jun(+)/BEX2-KD and c-Jun(+)/control-siRNA cells. Notably, we observed a significant increase in PP2A activity by 1.4 to 1.5-fold following BEX2-KD (Figure 5D). These findings suggest that BEX2 expression regulates PP2A activity in c-Jun(+) lines.

### There is a positive correlation between the expression of BEX2 and c-Jun in breast tumors

**Figure 6:**
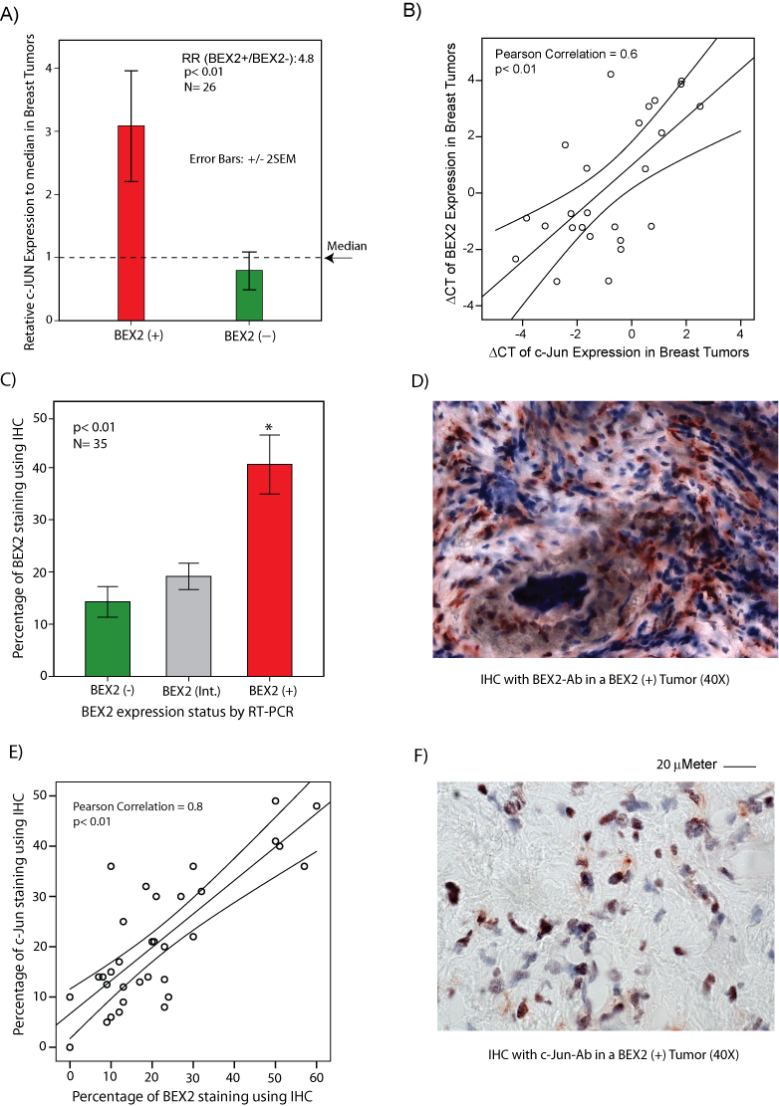
**The correlation of BEX2 and c-Jun expression in primary breast tumors. **(A) Relative c-Jun expression in BEX2 (+) and BEX2 (-) breast tumors using RT-PCR. RR: is relative c-Jun expression in BEX2(+)/BEX2(-). The expression differences between BEX2 over-expressed (BEX2 (+)) and BEX2 under-expressed (BEX2 (-)) samples are at least 3-fold. Error Bars: ± 2SEM. (B) Correlation between BEX2 and c-Jun gene expression. Scatter plot demonstrates the correlation of ΔCT values for BEX2 and c-Jun expression using RT-PCR in breast tumors. Correlation coefficient (0.6) is measured using Pearson's method. Linear regression line (best-fit) and 95% confidence interval lines are depicted (N = 26, p < 0.01). (C) BEX2 protein levels using immunohistochemistry (IHC). BEX2 staining was carried out using IHC with rabbit polyclonal BEX2 antibody at 1:50 dilution. Percentage of cells with BEX2 staining is compared between the following groups, which are previously defined by RT-PCR; BEX2 (+): ≥ 3-fold higher gene expression to median, BEX2 (-): ≥ 3-fold lower gene expression to median, and BEX2 (int.; intermediate): < 3-fold gene expression change to median. *, is for BEX2 (+) vs BEX2 (-) or BEX2 (Int.) groups. Error Bars: ± 2SEM. (D) BEX2 staining by IHC in a BEX2 (+) breast tumor at 40× magnification. (E) Correlation of BEX2 and c-Jun protein levels. Scatter plot demonstrates the correlation between the percentage of c-Jun and BEX2 staining using IHC in breast tumor samples. Rabbit c-Jun monoclonal antibody was used at 1:50 dilution. Correlation coefficient (0.8) is measured using Pearson's method. Linear regression line (best-fit) and 95% confidence interval lines are depicted (N = 35, p < 0.01). (F) c-Jun staining by IHC in a BEX2 (+) breast tumor at 40× magnification.

To further study our findings using actual breast cancer tissue, we investigated a correlation between the expression of BEX2 and c-Jun in primary breast tumors. We first assessed a possible correlation between the transcript levels of BEX2 and c-Jun in a cohort of 35 frozen breast tumors. BEX2 expression was measured using RT-PCR and normalized to the median expression of BEX2 across the cohort. In order to divide the cohort into two groups with either over- or under-expression of BEX2, we removed nine samples with a borderline BEX2 expression (BEX2-intermediate) so that the expression differences between BEX2 over-expressed (BEX2 (+)) and BEX2 under-expressed (BEX2 (-)) samples were at least 3-fold [2]. We next measured c-Jun expression in breast tumors using RT-PCR and normalized the data to the median expression of c-Jun across the cohort. Subsequently, we compared the level of c-Jun expression between BEX2 (+) and BEX2 (-) samples and found it to be markedly higher in BEX2 (+) tumors by approximately 4.8-fold compared to the BEX2 (-) samples (p < 0.01, Figure 6A). Furthermore, there was a Pearson's correlation coefficient (CC) of 0.6 between BEX2 and c-Jun transcript levels in this data set (p < 0.01, Figure 6B).

We next examined a correlation between BEX2 and c-Jun protein levels in breast tumors using immunohistochemistry (IHC). For this purpose we first optimized the rabbit polyclonal BEX2 antibody for IHC application on frozen breast tumors. We validated the quality of BEX2 antibody for this application by comparing the results of BEX2 staining using IHC with the BEX2 transcript levels using RT-PCR in the same cohort (Figure 6C-D, and Additional file 4, Figure S4). We observed that BEX2 (+) and BEX2 (-) tumors defined by RT-PCR had 44% (± 5) and 14% (± 3) BEX2 IHC staining, respectively (Figure 6C and 6D). In addition, BEX2 intermediate group defined by RT-PCR had 19% (± 3) BEX2 staining (Figure 6C and Additional file 4, Figure S4A). Notably, BEX2 protein level using IHC was significantly higher in BEX2 (+) group compared to the BEX2 (-) and BEX2-intermediate groups (p < 0.01), indicating that IHC and RT-PCR data correlate well in this cohort. Moreover, negative control experiments did not show any non-specific staining (Additional file 4, Figure S4B). Subsequently, we studied the correlation between BEX2 and c-Jun protein levels in these breast tumors using IHC. Importantly, we observed a strong correlation with a CC of 0.8 between the percentage of cells with BEX2 and c-Jun staining in this cohort (N = 35 and p < 0.01, Figure 6E and 6F). Taken together, these data indicate that there is a positive correlation between the expression of BEX2 and c-Jun in primary breast tumors.

## Discussion

We have previously demonstrated that BEX2 has a significant role in promoting cell survival and growth in breast cancer cells [1,2]. In this respect, BEX2 expression protects breast cancer cells against mitochondrial apoptosis and is necessary for the normal transition of these cells through G1 cell cycle [2]. In addition, it has recently been shown that down-regulation of BEX1 and BEX2 sensitize LNT-229 glioma cells to the chimeric tumor suppressor-1 (CST-1), a dominant-positive variant of p53, and up-regulation of BEX1 protects these cells to CST-1-induced cell death [22]. These findings further support a pro-survival function for BEX1 and BEX2 using a glioma model. Moreover, BEX2 is differentially expressed in breast tumors and is associated with a characteristic gene-expression signature in this disease [1]. Therefore, understanding the transcriptional regulation of BEX2 is a critical step to advance our knowledge about the function of this gene in the biology of breast cancer.

The available data in different cancers suggest that BEX2 expression can be regulated by a variety of mechanisms. Le Mercier et al. have recently reported that galectin 1, a key player in astroglioma and oligodendroglioma cell migration, has a regulatory effect on BEX2 expression in oligodendroglioma cells [23]. These authors have demonstrated that down-regulation of galactin 1 in oligodendroglioma cells results in a marked reduction of BEX2 expression [23]. Furthermore, decreasing BEX2 expression in these cells impairs neoangiogenesis and cell migration [23]. It is also notable that galactin 1 is up-regulated in breast cancer and has a possible role in tumor-stroma interaction in this disease [24]. Furthermore, in MLL wild-type AML and glioblastoma BEX2 expression is regulated by epigenetic silencing such as promoter methylation [6,8]. However, in MLL mutant AML cells there is a constitutive expression of BEX2 accompanied by promoter hypomethylation [8]. It is notable that in contrast to these cancer types, we have not found any correlation between BEX2 expression and promoter methylation in breast tumors [1]. Importantly, as opposed to the down-regulation of BEX2 expression observed in gliobalstoma there is a relative overexpression of this gene in breast tumors, which suggests a difference in the transcriptional regulation of BEX2 between these cancers [1,6]. Interestingly, BEX2 has a higher expression in low grade oligodendroglioma compared to glioblastoma and there are differences in the biological function of this gene between these tumor types [23], which suggest a variation in the transcriptional regulation and function of BEX2 in different brain malignancies.

In order to investigate the transcriptional regulation of BEX2, we first examined the factors involved in the regulation of BEX2 expression in breast cancer cells. We confirmed our previous observation that ceramide treatment has a striking effect on the induction of BEX2 expression and showed that this effect can be almost completely reversed using IкBα phosphorylation inhibitor BAY11 or the overexpression of IκBα-DN (Figure 1A). These findings suggested that transcription factors known to be activated by ceramide signaling and NF-κB activation are potentially involved in the transcriptional regulation of BEX2. Transcription factors c-Jun/AP-1 and AP-2 are known to be activated by the ceramide signaling pathway [13,14,25]. Coordinated induction of ceramide and c-Jun/JNK has an important role in stress-induced apoptosis[13,25]. In addition, ceramide induction of intercellular adhesion molecule-1 (ICAM-1) expression requires the activation of AP-2 through a cytochrome c-dependent mitochondrial pathway [14]. Furthermore, ceramide activates transcription factor NF-κB including both p65/RelA and p50/NF-κB1components of this protein complex [17,25]. Moreover, the bioinformatics analysis of BEX2 promoter identified several candidate binding sites for c-Jun/AP-1, NF-κB/p65, and AP-2 transcription factors on BEX2 promoter including six binding sites for c-Jun/AP-1 (Figure 1B). Importantly, we observed a significant induction of BEX2 promoter by 11-fold for c-Jun and by 2.7 to 5-fold for the other transcription factors (Figure 1C), providing strong experimental support for the bioinformatics analysis. In addition to showing a strong effect in the functional transcriptional assay, we also proved that c-Jun and p65/RelA are physically present at the BEX2 promoter with a panel of ChIP assays (Figure 1D and 1E). Moreover, there was a 2-fold increase in the observed enrichment by c-Jun antibody following ceramide treatment of MCF-7 cells (Figure 1F). A similar pattern of increase in enrichment following ceramide treatment has been reported with another c-Jun target gene Beclin1, which is also inducible by ceramide [26]. These findings demonstrate that BEX2 is a target gene of c-Jun and p65/RelA. Moreover, c-Jun has a clear role in the ceramide-mediated induction of BEX2 expression.

We have also demonstrated that the transcriptional regulation of BEX2 by c-Jun and p65/RelA translated through to BEX2 protein expression and we were able to show that there is a strong correlation between BEX2 and c-Jun expression levels in primary breast tumors. Moreover, we have previously demonstrated that p65-nuclear staining by IF is approximately 2-fold higher in primary breast tumor samples with a relative overexpression of BEX2 [2]. Overall, these findings demonstrate that BEX2 expression has a positive correlation with the expression of c-Jun and activation of p65 (nuclear) in primary breast tumors. These data using actual breast cancer tissue support our *in vitro *findings regarding the transcriptional regulation of BEX2 by c-Jun and p65/RelA. Furthermore, our findings suggest that the relative overexpression of BEX2 in a subset of breast tumors can be explained by a higher expression/activation of c-Jun and p65 transcription factors in this subset.

It has been shown that a number of c-Jun and p65/RelA target genes are involved in mediating the cellular functions of these proteins [27-29]. For example NF-κB induction of Bcl-2 is functionally linked to its pro-survival activity [28,29]. In addition, HMG-I/Y is involved in c-Jun mediated anchorage-independent growth and the activation of c-Jun/JNK pathway can mediate Beclin 1 expression, which plays a key role in autophagic cell death in cancer cells [26,27]. We were able to detect a similar feedback loop in the BEX2 system. There was a significant induction of p65 nuclear localization following BEX2 overexpression, which was inhibited using IкBα phosphorylation inhibitor BAY11 and BEX2-KD reversed a ceramide-mediated increase in p65 DNA binding. It is notable that the inhibitory effect of BAY11 on p65 activation was not overcome by BEX2 overexpression. This is likely due to the fact that IкBα phosphorylation is a necessary step in p65/NF-κB activation [30]. Moreover, our findings explain a possible mechanism underlying the observed effect of BEX2 expression on p65 activation, as there was a modest but reproducible reduction in p65 and IкBα phosphorylation following BEX2-KD. Overall, these findings indicate that BEX2 expression is required for the adequate activation and phosphorylation of p65 in an IкBα-dependent fashion. In addition, we observed similar functional effects of BEX2 expression in the regulation of c-Jun with striking reductions in c-Jun phosphorylation following BEX2-KD. This can be explained by our finding of marked reduction in JNK kinase activity following BEX2-KD. Since JNK is a key regulator of c-Jun phosphorylation, a reduction in JNK activity is a likely cause of the observed decrease in c-Jun phosphorylation level following BEX2-KD. Importantly, our data suggest that BEX2 regulates the phosphorylation of c-Jun and p65 at Ser63 and Ser468 sites, respectively. In turn, these phosphorylation sites are required for the effect of c-Jun and p65 in the transcriptional activation and binding to BEX2 promoter region. Taken together, these data show that the BEX2 pathway shares this feedback feature with some of the other c-Jun and p65/RelA target genes.

The functional data presented in this study suggest that BEX2 has a regulatory feedback loop with c-Jun and p65 signaling in breast cancer cells. Moreover, these findings are supported by a strong correlation between BEX2 and c-Jun expression patterns as well as a higher level of p65 activation associated with BEX2 overexpression in breast tumor samples [2]. Considering the importance of c-Jun and p65/NF-κB pathways in breast tumor development and progression [31,32], this feedback mechanism has significant biological implications in breast cancer.

To gain a deeper understanding of the effects of BEX2 expression in c-Jun-mediated cellular functions we investigated the effect of BEX2 on cyclin D1 which is a known c-Jun target of obvious importance in breast cancer. To do this, we generated two stable c-Jun(+) cell lines. These had higher expression of cyclin D1than control lines, and their cyclin D1 levels were markedly reduced by BEX2 knock-down. Cyclin D1 is a c-Jun target gene and is involved in c-Jun-mediated G1 progression [21,33]. In addition, we noted a significant decrease in the baseline cell growth and c-Jun-mediated induction of cell proliferation following BEX2-KD (p < 0.01, Figure 5C). These findings suggest that BEX2 expression is necessary for c-Jun-mediated induction of cyclin D1 and cell proliferation in breast cancer cells. Moreover, we have previously reported that BEX2 down-regulation in breast cancer cells leads to a G1 arrest and a significant reduction of cyclin D1 expression [2]. Considering the data presented here, the observed effects of BEX2 expression on G1 cell cycle and cyclin D1 can be a consequence of BEX2 regulation of c-Jun.

In this study, we demonstrate that BEX2 expression is required for the adequate phosphorylation of p65, IκBα, and c-Jun as well as JNK kinase activity. Importantly, these proteins are known to be directly regulated by PP2A [12,34-36]. Furthermore, we have recently shown that BEX2 regulates PP2A expression and activity in breast cancer cells [2]. Moreover, here we found a significant increase in PP2A phosphatase activity following BEX2 down-regulation in c-Jun(+) stable lines (Figure 5D). Overall, these findings provide a possible mechanism for the functional effects of BEX2 expression on p65, IκBα, and c-Jun/JNK through the regulation of PP2A activity.

## Conclusions

**Figure 7:**
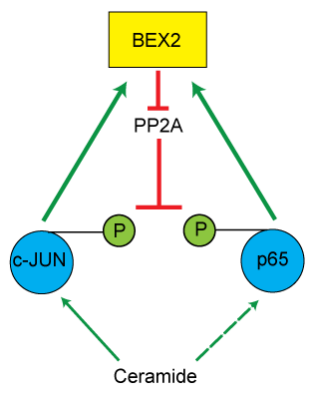
**Schematic diagram of BEX2 interplay with c-Jun and p65. **The diagram depicts BEX2 interactions with c-Jun, p65, PP2A, and ceramide. Green arrow: stimulatory effect; Red crossed-line: inhibitory effect. P: phosphorylated protein.

In summary, this study shows that BEX2 has a functional interplay with c-Jun and p65/RelA in breast cancer (Figure 7). In this feedback process BEX2 is a target gene for c-Jun and p65/RelA. BEX2 in turn regulates the phosphorylation of c-Jun, p65, and IκBα as well as JNK kinase activity in breast cancer cells. BEX2-mediated regulation of PP2A activity provides a possible mechanism for these functional effects. Our findings suggest that BEX2 is involved in a novel feedback mechanism with significant implications for the biology of breast cancer.

## Methods

### Cell culture and cell line treatments

Breast cancer cell lines MCF-7 and MDA-MB-231 were cultured in DMEM media (Invitrogen), 10% Fetal Bovine Serum (FBS). Treatments with ceramide analogue, C2 (Sigma) at 10 μM concentration, IкBα phosphorylation inhibitor BAY11-7082 (Merck) at 5 μM concentration, and beta NGF (R&D Systems) at 200 ng/ml concentration were carried out overnight in serum-free media.

### Real Time-PCR analysis in cell lines

Total RNA extraction was performed as described before [1]. RT-PCR to assess the expression level of BEX2 (assay ID: Hs00607718_g1) was carried out using Taqman^® ^Gene Expression Assays (Applied Biosystems) as instructed by the manufacturer. Housekeeping genes HPRT1 and RPLP0 (Applied Biosystems) were used as controls. Relative gene expression = gene expression in the treated group/average gene expression in the control group. All experiments were performed in four biological replicates.

### Bioinformatics analysis

The sequence of the 1 kb promoter region of BEX2 was obtained using Ensembl Genome Browser http://www.ensembl.org/index.html. Identification of putative transcription factor binding sites in the promoter region of BEX2 was carried out using PATCH™ public 1.0 software http://www.gene-regulation.com/cgi-bin/pub/programs/patch/bin/patch.cgi and TRANSFAC^® ^6.0 data base http://www.gene-regulation.com/cgi-bin/pub/databases/transpath/search.cgi. The data was then examined for the number and location of binding sites for each putative transcription factor.

### Site-Directed Mutagenesis

Mutant constructs of c-Jun (Ser63→Ala) and p65 (Ser468→Ala) were generated using QuikChange Site-Directed Mutagenesis Kit (Stratagene) following manufacturer's instructions. The mutagenic primers were designed using Stratagene QuikChange Primer Design Program http://www.stratagene.com/qcprimerdesign. The following mutagenic (m) primers were used: Jun(m)-forward: gacctcctcaccgcgcccgacgtgg, Jun(m)-reverse: ccacgtcgggcgcggtgaggaggtc, p65 (m)-forward: gtgttcacagacctggcagccgtcgacaact, p65(m)-reverse: agttgtcgacggctgccaggtctgtgaacac. The generated mutations in the selected clones were verified using sequencing.

### Luciferase Reporter assays

Full-length cDNA clones for c-Jun, p65/RelA, p50/NF-κB1, and AP2α were obtained from Open Biosystems (Thermo scientific). The clones were validated by restriction digestion/sequencing and then sub-cloned in pcDNA™3.1 vector (Invitrogen) to generate expression constructs. Furthermore, the sequence of 1.2 kb promoter region of BEX2 was obtained using Ensembl Genome Browser and PCR-generated using the following primers; (Forward-primer: ggcctaggatccattttgaa and Reverse-primer: gatcacgtgtggctgttgtc). Subsequently, BEX2 promoter was cloned in a pGL3 luciferase reporter vector (Promega) and validated by restriction digestion/sequencing. To carry out the reporter assays, MCF-7 cells were co-transfected with the BEX2 reporter vector and each of the transcription factors or mutant constructs of c-Jun (Ser63→Ala) and p65 (Ser468→Ala) using ExGen 500 reagent (Fermentas Life Sciences). The *Renilla *pRL-TK vector was used as an internal control reporter. Co-transfection with the BEX2 reporter vector and the empty pcDNA vector were used as the control. Forty-eight hours after the transfections reporter activities were measured using Dual-Glo™ Luciferase Assay System (Promega) in an Orion II Microplate Luminometer (Berthold Detection Systems). The response ratios for transcription factors and control were measured relative to the internal control reporter (relative response ratio). All reporter assays were carried out in eight biological replicates.

### ChIP Assays

Chromatin immunoprecipitation (ChIP) assays were performed in MCF-7 cell line using ChIP Assay Kit (USB Corporation) as instructed by the manufacturer. ChIP-grade rabbit polyclonal p65 (AbCam) and rabbit polyclonal c-Jun (Millipore) antibodies were applied for these assays at 1:100 and 1:50 dilutions, respectively. Sonication was carried out at 50% output for 8 cycles of 30 sec pulses with 2 min cooling in between each cycle. This process generated chromatin fragments with an average size of 200-500 bp assessed using Agarose gel electrophoresis. Four sets of primers for BEX2 promoter were used for the end point RT-PCR amplification using SYBR green method (Applied Biosystems). These included; Primer set1: Forward primer: caagcaggggaagtctcaag (start -136) and Reverse primer: ccgggagtcccttttaacat (start -57), Primer set 2: Forward primer: aggctggggatgttaaaagg (start -85) and Reverse primer: gatcacgtgtggctgttgtc (start +46), Primer set 3: Forward primer: gccctgtccttttccaagtt (start -551) and Reverse primer: aaatgtcccaaccacctgtc (start -462), and Primer set 4: Forward primer: gccctgtccttttccaagtt (start -868) and Reverse primer: cccaaccacctgtcctgtta (start -748). These primers were quality controlled using PCR amplification of MCF-7 genomic DNA followed by Agarose gel electrophoresis and sequencing. Amplification of input chromatin at a dilution of 1:100 prior to immunoprecipitation was used as a positive control for ChIP assays and ChIP using non-specific antibody (rabbit IgG) and distant primer sets (5 kb) served as negative controls. ChIP experiments were carried out with and without ceramide induction at 10 μM concentration overnight. The assays were carried out in four biological replicates and copy number changes were calculated as -Log2 value for each experimental set.

### Western blot analysis

Total-c-Jun rabbit monoclonal antibody, phospho-c-Jun (Ser63) rabbit monoclonal antibody, IκB-α rabbit polyclonal antibody, phospo-IκB-α (Ser32/36) mouse monoclonal antibody, and cyclin D1 rabbit monoclonal antibody were obtained from Cell Signaling, MA. Western blots with these antibodies were carried out at 1:1000 dilution of each primary antibody using 20 μg and 30 μg of protein lysates for total and phospho-antibodies, respectively. Western blot for p65 was performed with p65 rabbit polyclonal (AbCam) at 1:500 dilution using 30 μg of protein lysate. Furthermore, western blot analysis with rabbit polyclonal BEX2 antibody was performed at 1:200 dilution using 10 μg of protein lysate. This anti-BEX2 antibody was generated by us through Quality Controlled Biochemicals (MA) as describe previously [2]. Protein concentrations from the cell isolates were measured using the BCA Protein Assay Kit (Thermo scientific) and rabbit polyclonal α-tubulin antibody (Abcam) was used as the loading control. Analysis of band densities was performed using Bio-Profil Densitometer Software (Vilber Lourmat, Germany). All fold changes in band densities were measured relative to the control groups. Western blot experiments were carried out in three biological replicates and average fold changes are reported.

### Transient overexpression experiments

MCF-7 cells were grown to 60% confluence. Overexpression of BEX2 was performed using a BEX2 construct in pReciever expression vector (GeneCopoeia, MD) as described previously [2]. Overexpression of dominant-negative IκBα was carried out using the IκBα Dominant-Negative Vector Set (Clontech). The IκBα-DN vector contains a mutated form of IκBα with a serine-to-alanine mutation at residues 32 and 36. Overexpression of c-Jun and p65/RelA were carried out using the respective expression constructs in a pcDNA™3.1 vector (Invitrogen) as explained above. Mutant constructs c-Jun (Ser63→Ala) and p65 (Ser468→Ala) were generated using QuikChange Site-Directed Mutagenesis Kit (Stratagene) as described above and overexpressed in MCF-7 cells. Empty vectors were used as the negative controls. Transfection of MCF-7 cells was performed using ExGen 500 reagent (Fermentas Life Sciences), as instructed by the manufacturer. All experiments were performed in four biological replicates.

### BEX2 Knock-Down in cell lines

BEX2-Knock Down was carried out using two sets of siRNA Oligos (duplex), (Sigma-Genosys): Duplex 1/2: (D1: 5'rCrArGUrAUrArGrAUrGrGrGrArCrAUrArATT, D2: 5'UUrAUrGUrCrCr CrAUrCUrAUrArCUrGTT); Duplex 3/4: (D3: 5'rGrArGrCrGUUrArArArCrArAUrCUrCrAU TT, D4: 5'rAUrGrArGrAUUrGUUUrArArCrGrCUrCTT) as described before [2]. Transfection of siRNA oligos using Lipofectamine ™ RNAiMAX (Invitrogen) was carried out by reverse transfection method as instructed by the manufacturer. The final siRNA duplex concentration was 10 nM for all the knock-down experiments. Cells transfected with si*CONTROL*™ Non-Targeting siRNA, (Dharmacon Inc.) were used as controls. In all experiments the effects of BEX2-KD were assessed seventy-two hours after the siRNA transfections. BEX2-KD experiments were carried out separately with two siRNA oligos and the data presented for each knock-down experiment is the average result obtained from these two duplexes. All siRNA silencing experiments were performed in four replicates with each duplex.

### Immunofluorescence staining

Immunofluorescence (IF) staining in MCF-7 cells was performed as described previously [2]. IF staining was carried out 48 h after transfections to detect protein overexpression or at 72 h time point to assess the effect of chemical treatments with ceramide and BAY11. For primary antibodies BEX2 rabbit polyclonal [2], and p65 rabbit polyclonal (AbCam) antibodies were applied at 1:100 and 1:200 dilutions, respectively. Alexa-594 anti-rabbit secondary antibody (Invitrogen) was applied at 1:500 dilution. Scoring was performed in a total of 1000 cells for each slide using a confocal microscope (Carl Zeiss) with ZEN 2008 imaging software. To assess the nuclear localization, the percentage of cells which showed only nuclear staining pattern with p65-IF (Nuclear-Only staining) was calculated in each group. All experiments were performed in four biological replicates.

### ELISA Assays

#### 1) Phospho-p65 NF-кB

MCF-7 cells were grown in 96-well plates. Seventy-two hours after siRNA transfections, the amounts of phospho-p65 and total-p65 NF-кB proteins were measured using ELISA (CASE™ NF-кB p65 S468 kit, SA Biosciences) in BEX2-KD and siRNA-control groups. Experiments were carried out in four biological replicates and the ratio of phospho-p65/total-p65 was obtained for each experimental group.

#### 2) p65 NF-кB DNA Binding

Seventy-two hours after transfections nuclear extraction was carried out using Nuclear Extraction Kit (Panomics Inc., CA) and p65 NF-κB DNA binding in 10 μg of starting nuclear extract was measured by ELISA (NF-кB p65 ELISA Kit, Panomics Inc, CA). The experiments were carried out in the following groups: 1) control-siRNA, 2) control-siRNA + ceramide treatment at 10 μM overnight, 3) BEX2-siRNA + ceramide, 4) control-vector + BAY11 at 5 μM ON, and 5) BEX2-vector + BAY11. Four biological replicates were performed for each group.

### JNK Kinase Assay

JNK kinase assay was carried out using JNK Assay Kit (Cell Signaling) following manufacturer's instructions and as described before [37]. This assay was performed by a selective immunoprecipitation (IP) of JNK using immobilized c-Jun fusion protein to Agarose beads followed by the incubation of IP pellets in Kinase Buffer containing cold ATP. The assay was then analyzed using western blot with phospho-c-Jun (Ser63) rabbit monoclonal antibody (Cell Signaling) at 1:1000 dilution. Ceramide treatment at 10 μM concentration overnight was used as a positive control. Fold changes in band densities were measured relative to the control group. Experiments were carried out in three biological replicates and average fold changes are shown.

### Generating c-Jun stable lines

MCF-7 cells were transfected with c-Jun/pcDNA™3.1vector as described above. Transfection with an empty vector was used as a control. To obtain stable c-Jun expressing clones, the transfected MCF-7 cells were selected in the presence of Geneticin^® ^Selective Antibiotic (Invitrogen) at 500 μg/ml concentration as instructed by the manufacturer. Single neomycin-resistant clones were picked and cultured in the presence of Geneticin at 200 μg/ml concentration as described before [38].

### MTT Assay

Stable c-Jun(+) clones and vector control were cultured in 96-well plates. BEX2-KD using reverse transfection method was carried out as explained before. Seventy-two hours after transfections, cell proliferation was assessed for BEX2-KD and control-siRNA experiments using Vybrant^® ^MTT Proliferation Assay Kit (Invitrogen) as instructed by the manufacturer. Absorbance at 570 nM was measured for all the experimental groups using a plate reader. MTT assays were performed in eight biological replicates.

### PP2A Assay

Cell lysis was carried out in lysis buffer deprived of phosphatase inhibitors as described before [39]. PP2A assay was carried out using PP2A Immunoprecipitation Phosphatase Assay Kit (Millipore), and pmoles of phosphate were measured for each group. Experiments were carried out in four biological replicates.

### Primary breast tumors

The institutional research ethics committee approved this study and informed consent was obtained from each patient for the use of tissue samples. A total of thirty-five frozen tumor samples were obtained from the Princess Alexandra Hospital tissue bank. Total RNA extraction from the frozen breast tumor samples was performed as we previously described [40]. RT-PCR to measure the expression of BEX2 and c-Jun (assay ID: Hs99999141_s1) was carried out using Taqman^® ^Gene Expression Assays (Applied Biosystems) as described above for the cell lines. Five-micron thick sections of frozen tumors were prepared for IHC using Cryostat (Leica Microsystems). IHC staining was performed using EnVision^®^+ System-HRP (AEC), (DakoCytomation) following manufacturer's instruction. Primary antibody incubations were carried out with BEX2 rabbit polyclonal and c-Jun rabbit monoclonal (Cell Signaling) antibodies at 1:50 dilutions. Hematoxylin was used as a counterstain. For IHC scoring each sample was examined using a light microscope (Nikon Instruments Inc.). A total of 800 cells per tumor sample were counted at 60× magnification and the percentage of cells showing BEX2 or c-Jun staining was calculated for each tumor.

### Statistical Analysis

Biostatistical analysis was done using the Statistical Package SPSS^® ^version 17.0 (Chicago, IL). Mann-Whitney U test was applied for the comparison of non-parametric data.

## Competing interests

The authors declare that they have no competing interests.

## Authors' contributions

AN conceived the study, performed the data analysis, and drafted the manuscript. AN and JL carried out the experiments. LHD contributed with scientific discussion and manuscript preparation. All authors read and approved the final manuscript.

## Supplementary Material

Additional file 1 **Figure S1. **Agarose Gel Electrophoresis for ChIP assay primer sets. Four primer sets for BEX2 promoter were quality controlled using PCR amplification of MCF-7 genomic DNA before application for ChIP assays. Agarose gel electrophoresis shows unique products with these primer sets. Click here for file

Additional file 2 **Figure S2. **ChIP to assess the binding of c-Jun and p65 mutants to BEX2 promoter. ChIP assays with c-Jun and p65 antibodies following the transient transfections of MCF-7 cells with either wild type c-Jun and p65/RelA or the mutant constructs of c-Jun (Ser63→Ala) and p65 (Ser468→Ala). Transfection with an empty pcDNA vector was used as a control. ChIP assays were carried out forty-eight hours after the transfections and the enrichment of BEX2 promoter region was assessed using the end point RT-PCR amplification with primer set 1 (see methods). Amplification of input chromatin at a dilution of 1:100 prior to immunoprecipitation was used as a positive control and ChIP using non-specific antibody (rabbit IgG) and distant primer sets (5 kb) served as negative controls. Copy number changes of end point RT-PCR amplification are shown as -Log2 value for each experimental set. *, is compared to the negative control. Error Bars: ± 2SEM. Click here for file

Additional file 3 **Figure S3. **Morphology of c-Jun(+) stable clones. (A) and (B): Images of control-vector MCF-7 line using Leica DM IL inverted microscope at 10× and 20× magnifications, respectively. (C) and (D): Images of stable c-Jun (+)-MCF-7 line at 10× and 20× magnifications, respectively. Click here for file

Additional file 4 **Figure S4. **BEX2 immunohistochemistry and negative control. (A) BEX2 staining using immunohistochemistry (IHC) in a sample with BEX2-intermediate expression (< 3-fold gene expression change to median). IHC was carried out with rabbit polyclonal BEX2 antibody at 1:50 dilution. Image is at 20× magnification. (B) Negative control for IHC with 2^nd ^antibody only staining. Image is at 20× magnification. Click here for file
