# Supplementary figures and images for: BEX2 has a functional interplay with c-Jun/JNK and p65/RelA in breast cancer
Source: Mol Cancer. 2010 May 19;9:111. doi: 10.1186/1476-4598-9-111 (PMC2881879; doi:10.1186/1476-4598-9-111)

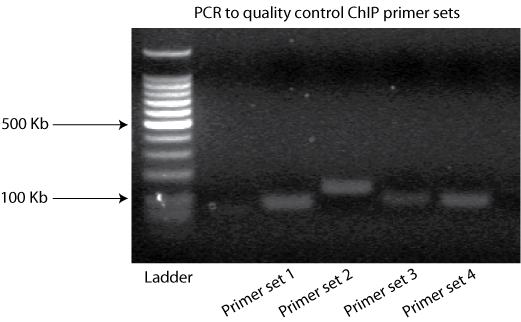

Supplement: Additional file 1 — Figure S1. Agarose Gel Electrophoresis for ChIP assay primer sets. Four primer sets for BEX2 promoter were quality controlled using PCR amplification of MCF-7 genomic DNA before application for ChIP assays. Agarose gel electrophoresis shows unique products with these primer sets. [file 1476-4598-9-111-S1.TIFF]

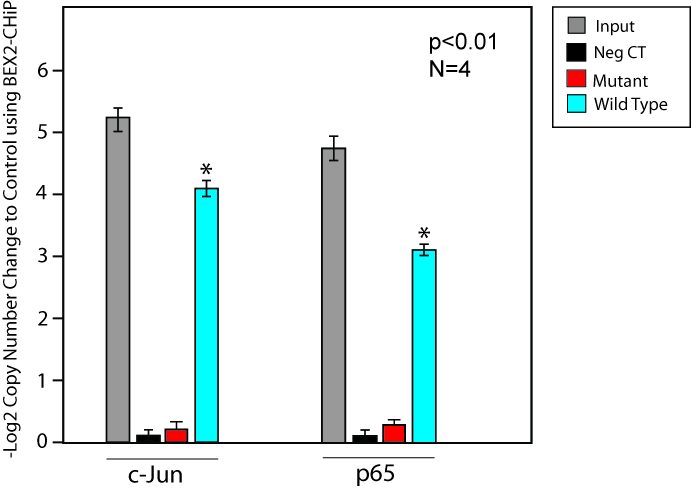

Supplement: Additional file 2 — Figure S2. ChIP to assess the binding of c-Jun and p65 mutants to BEX2 promoter. ChIP assays with c-Jun and p65 antibodies following the transient transfections of MCF-7 cells with either wild type c-Jun and p65/RelA or the mutant constructs of c-Jun (Ser63→Ala) and p65 (Ser468→Ala). Transfection with an empty pcDNA vector was used as a control. ChIP assays were carried out forty-eight hours after the transfections and the enrichment of BEX2 promoter region was assessed using the end point RT-PCR amplification with primer set 1 (see methods). Amplification of input chromatin at a dilution of 1:100 prior to immunoprecipitation was used as a positive control and ChIP using non-specific antibody (rabbit IgG) and distant primer sets (5 kb) served as negative controls. Copy number changes of end point RT-PCR amplification are shown as -Log2 value for each experimental set. *, is compared to the negative control. Error Bars: ± 2SEM. [file 1476-4598-9-111-S2.TIFF]

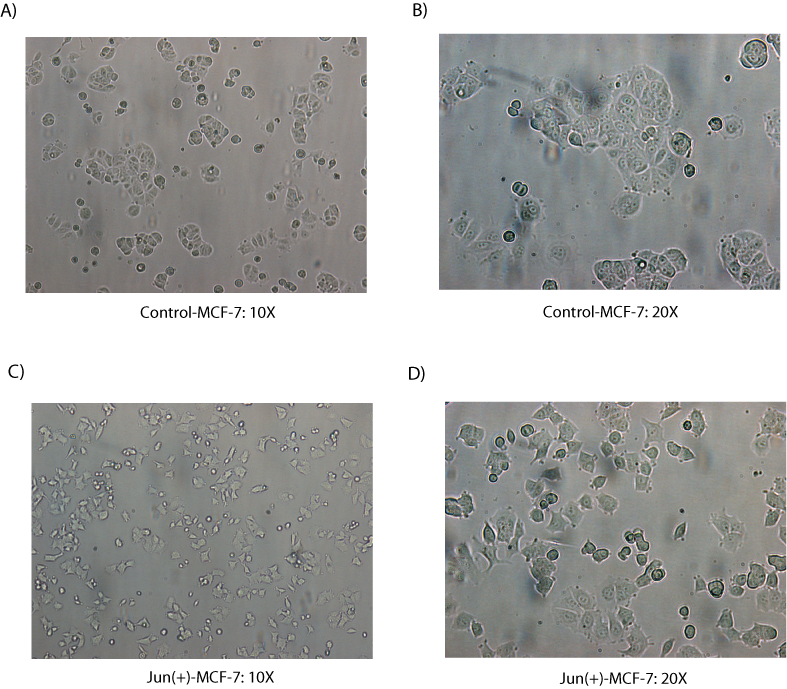

Supplement: Additional file 3 — Figure S3. Morphology of c-Jun(+) stable clones. (A) and (B): Images of control-vector MCF-7 line using Leica DM IL inverted microscope at 10× and 20× magnifications, respectively. (C) and (D): Images of stable c-Jun (+)-MCF-7 line at 10× and 20× magnifications, respectively. [file 1476-4598-9-111-S3.TIFF]

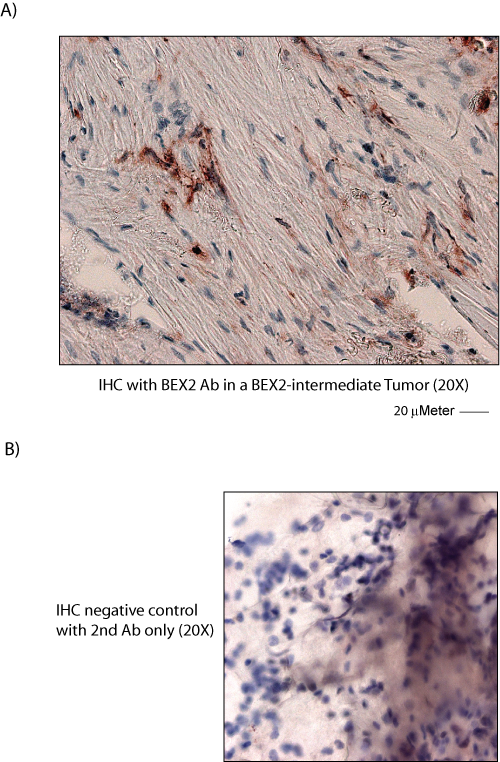

Supplement: Additional file 4 — Figure S4. BEX2 immunohistochemistry and negative control. (A) BEX2 staining using immunohistochemistry (IHC) in a sample with BEX2-intermediate expression (< 3-fold gene expression change to median). IHC was carried out with rabbit polyclonal BEX2 antibody at 1:50 dilution. Image is at 20× magnification. (B) Negative control for IHC with 2nd antibody only staining. Image is at 20× magnification. [file 1476-4598-9-111-S4.TIFF]
